# Development of quality indicators for hypertension management at the primary health care level in South Africa

**DOI:** 10.1038/s41371-024-00966-7

**Published:** 2024-10-14

**Authors:** Enos Muisaphanda Rampamba, Johanna Catharina Meyer, Brian Godman, Ntodeni Norah Ndwamato, Stephen Mark. Campbell

**Affiliations:** 1 Department of Public Health Pharmacy and Management, School of Pharmacy, Sefako Makgatho Health Sciences University, Pretoria, South Africa; 2 Division of Pharmacy Education, Professional Affairs, South African Pharmacy Council, Pretoria, 0001 South Africa; 3 Department of Pharmacoepidemiology, Strathclyde Institute of Pharmacy and Biomedical Sciences, University of Strathclyde, Glasgow, United Kingdom; 4 Department of Family Medicines, School of Medicine, University of Limpopo, Polokwane, South Africa; 5 Limpopo Department of Health, Limpopo Polokwane, South Africa; 6 Centre for Epidemiology and Public Health, School of Health Sciences, University of Manchester, Manchester, United Kingdom

**Keywords:** Diseases, Health care

## Abstract

Despite many quality initiatives at the primary health care (PHC) level, little is known about the actual quality of care of patients diagnosed with hypertension in South Africa. This study aimed to develop quality indicators for hypertension management at the PHC level to improve the quality of care and patient outcomes. The RAND/UCLA Appropriateness Method, comprising two rounds, was used to develop clear, appropriate, and feasible evidence-based quality indicators for hypertension. In Round 1, a 9-point scale was used by a panel of 11 members to rate clarity and appropriateness of 102 hypertension quality indicator statements, grouped under 9 dimensions of quality hypertension management, using an online MS Excel® spreadsheet. In Round 2, 9 of the same panellists discussed all indicators and rated their appropriateness and feasibility during a remote online, interactive face-to-face MS Teams® meeting. Statements rated ≥7–9 with agreement were defined as either appropriate or feasible. The panel rated 46 hypertension quality indicator statements ≥7–9 with agreement for the appropriate *and* feasible measurement of the management of hypertension: monitoring (*n* = 16), review (*n* = 5), lifestyle advice (*n* = 9), tests (*n* = 7), intermediate outcomes (*n* = 6), referrals (*n* = 2) and practice/facility structures (*n* = 1). No indicator statements were rated both appropriate and feasible for measuring blood pressure levels and treatment. If applied, these indicators would improve monitoring and management of patients with hypertension, patient outcomes, and data quality in South Africa and result in more efficient use of scarce resources. This study can be replicable for improving care of other non-communicable diseases across Africa.

## Introduction

South Africa has implemented multiple initiatives to improve the quality of the management of non-communicable diseases (NCDs), specifically hypertension and type 2 diabetes mellitus [1], at the primary health care (PHC) level covering approximately 80% of the population [2]. Consequently, compliance with the provision in the Constitution of the country [3]. This includes a National Strategic Plan for the Management of Non-Communicable Diseases, 2022-2027 [1]. Alongside this, policy and guidelines on monitoring and enforcing adherence to medicines for chronic use in all health facilities have been introduced [4]. In 2021, a National User Guide on the Prevention and Treatment of Hypertension in Adults at PHCs was rolled out. This included interventions to strengthen the monitoring and management of patients with hypertension in the country [5]. Recently, the National Health Insurance Bill paved the way to universal health coverage for all citizens in South Africa [6, 7]. Such interventions are important due to rising rates of NCDs in South Africa as well as an increase in the number of patients with co-morbidities, including both infectious diseases and NCDs [8, 9].

What remains unknown is the quality of care that patients with hypertension are currently receiving at the PHC level in South Africa [1, 10, 11]. It has been suggested that patients with diagnosed hypertension are not receiving recommended care and are often not at target treatment goals. This is exacerbated by possible suboptimal quality of healthcare services at the PHC level [8] and often unavailable antihypertensive medicines within PHC facilities [12]. However, there are not enough reliable data available on the quality of care that patients with hypertension receive at the PHC level in South Africa. As a result, the quality of care currently provided cannot be fully assessed to guide the implementation of policy initiatives for quality improvement [13]. Poor quality of care, coupled with a lack of data to improve care, contribute to avoidable high morbidity and mortality due to hypertension [14]. A summary of what is already known about hypertension management in the public PHC level in South Africa, and the gaps in knowledge this study addresses is presented in the discussion.

Quality indicators measure whether the evidence-based guidelines regarding the current management of hypertension are being implemented [13] and can provide data to monitor the achievement of programme outcomes [15–17]. However, currently in South Africa there is no agreed minimum set of indicators to assess the quality of care and to monitor progress towards attainment of the hypertension objectives set out in these initiatives [15]. Developing and testing quality indicators as part of a multiple approach is key to strengthening health programmes and enhancing the quality and efficiency of clinical services [11, 18, 19]. This has implications not only for the management of hypertension in South Africa but also across Africa, given current concerns [20]. Consequently, the aim of this study was to develop evidence-based quality indicators for hypertension management and monitoring at the PHC level for South Africa.

## Materials and method

This study used a modified RAND/UCLA Appropriateness Method (RAM), initially developed by RAND Heath staff in collaboration with clinicians at the University of California in Los Angeles (UCLA) [21]. The RAM is an internationally recognized formal group consensus modified Delphi technique that allows for the development of consensus among experts where no previous consensus exists, with individual opinions forming a refined, aggregated and group opinion. This methodology has been used extensively, for example, in the UK for the construction of a patient safety toolkit for general medical practice [22]. It uses a series of sequential steps involving evidence synthesis, clinical scenario or indicator development, panellist selection, two-round rating process and the analysis of the results.

### Synthesis of possible quality indicators

The authors, co-ordinated by the lead author (EMR), conducted a rapid evidence assessment to review the international evidence-based clinical guidelines as well as national action plans for the management of chronic NCDs, specifically hypertension. Whilst more than five hypertension guidelines were  identified in the review, only five principal ones were referenced for the purpose of this article to provide a robust and succinct baseline. The purpose was to identify, synthesise and develop quality indicator statements from international and national evidence-based guidelines for the management of diagnosed hypertension in adult patients. The indicator statements were identified and categorised into different dimensions of the quality of care from the quality standards guidelines for the management of hypertension at the PHC level, sourced from international and national guidelines and the National Strategic Plan for Non-communicable Diseases in South Africa (see Table 1) [1, 5, 23–25].

**Table 1:** Key sources of quality indicator statements.

Source	Description	Relevance to the development of quality indicators for hypertension management at the primary health care level in South Africa
National Department of Health (NSP). 2022. National strategic plan for the prevention and control of non-communicable disease 2022-2027. Available from: https://bhekisisa.org/wp-content/uploads/2022/06/NCDs-NSP-SA-2022-2027-1.pdf	The NSP sets the targets for the number of adults that have raised blood pressure who should receive interventions (60%), and those receiving interventions that should be controlled (60%).	The prescribed targets in the NSP are important in determining what type of indicators should be developed and tested in the South African PHC settings to realise the NSP.
NICE quality and outcomes framework Indicators 2020: Available from:	NICE indicator framework details how to develop and implement quality indicators and provide examples of quality indicators in the management and monitoring of hypertension.	RAND/UCLA Appropriateness Method was used to develop the NICE indicator framework. These indicators can also be tested and tailor-made to suit the South African PHC level using the same method.
National Department of Health. 2021. National User Guide on the prevention and treatment of hypertension in adults at the PHC level 2021. Available from: https://www.knowledgehub.org.za/system/files/elibdownloads/2021-11/HYPERTENSION%20USER%20GUIDE%20FINAL%20COPY.pdf	It is intended for use by PHC professionals to ensure that patients with confirmed diagnosis of hypertension receive evidence-based treatment and care.	It is important for this study as it provides the domains in the management of patients with confirmed hypertension at the PHC.
Pan American Health Organisation (PAHO). Monitoring and evaluation framework for hypertension control programs. Washington, DC. 2018. https://iris.paho.org/bitstream/handle/10665.2/34877/PAHONMH18001_eng.pdf?sequence=6&isAllowed=y	This is a collaboration between the Pan American Health Organization and the World Hypertension League that provides a broad international approach to a monitoring and evaluation framework for hypertension control programs.	It provides international examples of quality indicators for the management and monitoring of hypertension, that can be tested for suitability to the PHC settings in South Africa
National Department of Health.2020. Standard Treatment Guidelines and Essential Medicine List for South Africa. Available from: https://www.knowledgehub.org.za/system/files/elibdownloads/2021-02Primary%20Healthcare%20STGs%20and%20EML%207th%20edition%20-%202020-v2.0pdf	PHC is the first level of care at which most patients access the health system. In addition to these treatment guidelines aimed at healthcare workers at PHC facilities, the Essential Medicines List Clinical Guide mobile application encourages improved access to Standard Treatment Guidelines at all levels of care.	This guideline is important for this study as it lays the foundation of how PHC services are provided, and it emphasises the provision of evidence-based treatment and hence the importance of developing quality indicators to measure compliance in the use of this process.

### RAND/UCLA Appropriateness Method (RAM)

The RAM was used to identify the clarity, appropriateness and feasibility of the quality indicator statements that were derived from the literature review as part of an ongoing indicator development and testing protocol [26]. Panellists for the RAM were selected to create a multidisciplinary panel that reflected the range of health care professionals (HCPs) currently involved in the day-to-day management of people with diagnosed hypertension in South Africa. In addition, to contain a mixture of international/national experts along with other HCPs working in PHC facilities in South Africa. Panellists were recruited using nominations from professional associations, national, provincial and districts health departments and institutions of higher learning, including tertiary hospitals.

All panellists were involved in both rounds of the RAM. Panellists were sent a copy of the synthesised evidence synthesis, an instruction sheet including definitions of terms and the rating scale before completing the two appropriateness rating rounds. In both rounds, the panellists had the same number of hypertension quality indicator statements, grouped within 9 dimensions for their consideration and rating, based on the different sections of hypertension management guidelines nationally and internationally [1, 5, 23–25]. The dimensions consisted of “monitor, review, lifestyle advice, blood pressure levels, treatment, tests, intermediate outcomes, referrals and practice/facility” quality indicator statements. These dimensions are aligned to the sub-sections of the hypertension guidelines, for example, the section on treatment and monitoring of hypertension management as well as referral of patients within different levels of care.

In applying the rating scale, panellists were instructed to consider an adult patient (≥18 years) with a confirmed diagnosis of hypertension being managed by an ‘average clinician under average circumstances in the PHC setting in South Africa’.

Definitions used for clarity, appropriateness and feasibility in the context of this study were as follows:

Clarity meant that the indicator wording is clear and precise with unambiguous language [26]. Appropriate was defined as whether something would be an appropriate next step clinically for the ‘average’ patient in the ‘average’ PHC/ambulatory setting, seeing the ‘average’ HCP in South Africa with a rating of 7-8 [24, 26]. Necessary care was defined as an appropriate next step, without exceptions. Feasibility was defined as whether something would be feasible to implement, and data would be available for the ‘average’ patient in the ‘average’ PHC/ambulatory setting seeing the ‘average’ HCP, in terms of human resources, financial and other restraints in the context of South Africa [24, 26].

This study used the RAM which promotes robust, credible, and valid hypertension quality indicators, as this methodology combines the available scientific evidence and expert opinion in the management of hypertension [27, 28]. It is a practical, real-world method, designed to identify appropriate clinical steps tailored to patient needs and grounded in everyday practice of practitioners and facilities [21]. Unlike other consensus techniques such as the Delphi Technique, the RAM incorporates interactive discussion of indicator statements between panellists in Round 2. Online RAM panel meetings have been used successfully, in part in response to restrictions imposed by the COVID-19 pandemic, with online meetings at a reduced cost [28].

### Consensus procedure

The RAM was completed in two rounds. The first round consisted of an online MS Excel® rating spreadsheet that was sent to the panellists by email. The email requested them to rate the list of hypertension quality indicator statements on a 9-point integer scale, separately for clarity and appropriateness. Panellists were invited to provide alternative wording for the indicators or suggest new indicators if wished. The second round was an online face-to-face MS Teams® meeting chaired by two chairpersons: a clinical chairperson (NNN) and the methodologist chairperson (SMC) with experience in the use of consensus methods in different settings [27].

An MS Excel® spreadsheet was used to collate data from the completed first-round rating sheets with the median rating for each indicator calculated for clarity and for appropriateness. The frequency distribution of each response on the 1–9 scale for each indicator for clarity and appropriateness was calculated.

For Round 2, first-round data were used to create personalised rating sheets for each panellist showing the median for each indicator for clarity and appropriateness and the frequency distribution of all panel ratings across the 9-point integer scale for each indicator. This enables each panellist to see the first-round ratings of appropriateness from the full panel (confidentially to each panellist) and a reminder of how they rated each indicator in Round 1. Five days prior to the online meeting, panellists were sent their personalised rating sheet, an instruction sheet, source of evidence for the quality indicator statements, a summary of Round 1 and an introduction presentation explaining the RAM and purpose of Round 2. With agreement from all panellists, the panel was sent a short biography of each panellist.

The two chairs (SMC and NNN) of the second-round face-to-face online meeting also received an MS Excel® rating spreadsheet, showing how each panellist had rated each indicator in Round 1. In Round 2, panellists rated the indicators for appropriateness again and for feasibility in terms of the PHC level settings in South Africa using a 9-point scale for appropriateness and feasibility (See Table 2 for an explanation of the 9-point rating scale and panel rating scale consensus).

**Table 2:** 9-Point rating scale for appropriateness and feasibility.

Rating score (Number)	Description of the rating score
1	Unnecessary/Infeasible with no exceptions ever
2	Inappropriate/infeasible with some exceptions
2–3	Inappropriate/infeasible with general exceptions
4–6	Equivocal
7	Appropriate/feasible with general exceptions
8	Appropriate/feasible with some exceptions
9	Necessary/Feasible with no exceptions ever.
**Panel rating scale consensus**	
**Score (Number)**	**Description of score**
1–3	Inappropriate/ infeasible
4–6	Equivocal
7–9	Appropriate/ feasible.

An MS Excel® spreadsheet was used to collate data from the completed second-round rating sheets with the median rating for each indicator calculated for appropriateness and for feasibility. The frequency distribution of each response on the 1–9 scale for each indicator was calculated for appropriateness and feasibility.

### Data analysis

In both rounds of the RAM, the level of agreement for each indicator was calculated according to the conventional RAM method of percentage of ratings within the same tertile as the median (<25% as ’agreement’; ≥33% in both 1–3 and 7–9 ranges as ’disagreement’; all indicators without consensus [either agreement or disagreement] as ’equivocal’)’ [21, 29].

All data analyses and findings are based on Round 2 of the RAM.

## Results

### Panel composition

The panel was composed of 11 members in Round 1 of the RAM. Round 2 was composed of 10 members although one panellist failed to submit their rating scores resulting in a panel of 9 members. These included four family physicians, one cardiologist, one pharmacist, two clinical pharmacists and one clinical nurse practitioner. All panellists were involved in the management of hypertension patients and  contributing to development and review of hypertension guidelines, either at an international, national, provincial or district level.

### Quality indicator statements

There were 102 quality indicator statements listed and grouped under the 9 different domains of quality of care on the MS Excel® rating spreadsheet. No panellist provided alternative wordings for the indicators or suggested new indicators. The full list containing all 102 statements is available on request from the authors.

Table 3 summarises the results of the Round 2 indicator ratings with 46 quality indicator statements for hypertension (45.1%) rated appropriate (overall panel rating 7-8-9) and feasible (overall panel rating 7–9). None (0%) were rated with disagreement and 46 (45.1%) were rated equivocal for either or both scales. Of the 46 indicators rated appropriate, 10 (9.8%) were rated as a necessary next step without exceptions. For all indicators on both scales, there was agreement that no action was inappropriate (overall panel rating 1-2-3).

**Table 3:** Summary of appropriate and feasible ratings for round 2 RAM.

	Indicatotor domains									
	Monitor	Review	Lifestyle advice	Blood pressure	Treatment	Tests	Intermediate outcome	Referrals	Practice-facility	Total/Average
Indicators (n)	23	13	9	4	17	11	6	8	11	102
*Appropriate (%)*	95.7	61.5	100	100	82.4	90.9	100	100	100	
Median 1–3 (n)	0	0	0	0	0	0	0	0	0	0
Median 4–6 (n)	1	4	0	0	0	1	0	0	0	6
Median 7–9 (n)	22	9	9	4	17	10	6	8	11	96
Agreement (%)	100	69.2	100	100	82.4	100	100	100	100	94.6
Equivocal (%)	0	30.8	0	0	17.6	0	0	0	0	5.4
Disagreement (%)	0	0	0	0	0	0	0	0	0	0
*Feasible (%)*	69.6	76.9	100	0	0	63.6	100	25	27.2	51.4
Median 1–3 (n)	0	0	0	0	0	0	0	0	0	0
Median 4–6 (n)	4	2	0	3	11	2	0	0	4	26
Median 7–9 (n)	19	11	9	1	6	9	6	8	7	76
Agreement (%)	73.9	84.6	100	75	5.9	72.7	100	25	27.3	62.7
Equivocal (%)	26.1	15.4	0	25	94.1	27.3	0	75	72.7	37.3
Disagreement (%)	0	0	0	0	0	0	0	0	0	0

Key: Only statements rated ≥ 7 with agreement were considered appropriate or feasible

Only statements rated ≥7 with agreement were considered appropriate or feasible (Table 3). Under appropriateness, there were two statements rated 6 with agreement, and one statement rated 5 with agreement. Under feasibility, there were 9 statements rated 6 with agreement. There were 7 and 42 statements rated equivocal under appropriateness and feasibility respectively.

Table 4 shows the list of 46 statements for which the panel reached consensus in terms of their appropriateness and feasibility for hypertension management at the PHC level. Panellists did not find a single indicator being appropriate and feasible for the dimensions ‘patient blood pressure and patient treatment’. All the indicators about ‘advice about lifestyle and intermediate outcome’ were rated as appropriate and feasible. Most of the indicators under the dimensions ‘monitoring’ and ‘review of patients’ and indicators under the dimension ‘tests’ were found appropriate and feasible.

**Table 4:** List of hypertension management indicators rated appropriate and feasible.

Dimension	No.	Indicator	A	F
Patient monitoring	1.	The patient has a blood pressure recorded in their medical record in the last 12 months	9	8
	2.	The % of patients in the practice/unit/facility with a blood pressure recorded in the last 12 months	9	8
	3.	The patient has BMI recorded in their medical record in the past 12 months (A-8/F-8)	8	8
	4.	The patient has serum potassium concentration recorded in their medical record in the past 6 months for patients on spironolactone or eGFR <30 ml/min	9	7
	5.	The patient has serum creatinine concentration and eGFR recorded in their medical record in the past 12 months for patients with proteinuria of 1+ or more	8	8
	6.	The patient has serum creatinine concentration and eGFR recorded in their medical record in the past 12 months for patients with existing cardiovascular disease	8	7
	7.	The patient has serum creatinine concentration and eGFR recorded in their medical record in the past 12 months for patients with hypertension for 10 years or more	7	7
	8.	The patient has serum creatinine concentration and eGFR recorded in their medical record in the past 12 months for patients with uncontrolled hypertension	8	8
	9.	The patient has serum creatinine concentration and eGFR recorded in their medical record in the past 12 months for patients with chronic kidney disease (eGFR <60 mL/min)	9	8
	10.	The patient has fingerpick blood glucose recorded in their medical record in the past 12 months	9	8
	11.	The patient has urine protein by dipstick recorded in their medical record in the past 12 months	9	8
	12.	The % of patients in the practice/unit/facility who were screened for cardiovascular disease risk factors in the last 12 months	9	7
	13.	The % of patients in the practice/unit/facility who were checked for medicines and lifestyle modification adherence before escalating therapy	9	7
	14.	The % of patients in the practice/unit/facility who has urine protein by dipstick recorded in their medical record in the past 12 months	9	7
	15.	The % of patients in the practice/unit/facility who has serum creatinine concentration and eGFR recorded in their medical record in the past 12 months for patients with hypertension for 10 years or more	9	9
	16.	The percentage of patients aged 40 years and over with a blood pressure measurement recorded in the preceding 5 years	9	7
Patient review	17.	The patient has a hypertension review with a doctor recorded in their medical record in the last 12 months	9	8
	18.	The patient has a hypertension review with a nurse/doctor recorded in their medical record in the past 6 months after the BP is controlled, for patients who were having uncontrolled BP	7	7
	19.	The patient has a hypertension review with a nurse/ doctor recorded in their medical record one month after being in step 7 of the algorithm of hypertension management for patients already on medication	9	8
	20.	Number of those patients with a new diagnosis of hypertension aged 18–84 years, recorded (excluding those with pre-existing CHD, stroke and/or TIA), who have a recorded CVD risk assessment score of more than 20% in the preceding 12 months: the percentage who are currently treated with statins (unless there is a contraindication)	8	7
	21.	The percentage of patients with hypertension aged 18–74 years in whom there is an annual assessment of physical activity, in the preceding 15 months	7	7
Patient lifestyle advice	22.	The % of patients in the practice/unit/facility who has been counselled about the importance of smoking cessation in the last 12 months	9	7
	23.	The % of patients in the practice/unit/facility who has been counselled about the importance of maintaining ideal body weight, i.e. BMI < 25 kg/m^2^ in the last 12 months	9	7
	24.	The % of patients in the practice/unit/facility who has been counselled about the importance of salt restriction with increased potassium intake from fresh fruits and vegetables in the last 12 months	9	8
	25.	The % of patients in the practice/unit/facility who has been counselled about the importance of reducing alcohol intake to no more than 2 standard drinks per day for males and 1 for females, in last 12 months	9	7
	26.	The % of patients in the practice/unit/facility who has been counselled about to follow a healthy eating plan, in the last 12 months	9	7
	27.	There is evidence in the patient record that the nurse/ doctor counselled the patient on how and about the importance of engaging in physical activity, eat small portions of healthy food, use less salt, use alcohol in moderation, stop smoking, reduce stress, commit to take medication regularly	9	7
	28.	The % of patients in the practice/unit/facility who has been counselled about the importance of engaging in regular moderate aerobic exercise, e.g. 40 min brisk walking at least 3 times a week., in the last 12 months	9	7
	29.	The % of patients diagnosed with hypertension who are given lifestyle advice in the preceding 12 months for: smoking cessation, safe alcohol consumption and healthy diet (A-9/F-7)	9	7
	30.	The percentage of patients with hypertension and a BMI of 27.5 kg/m^2^ or more (or 30 kg/m^2^ or more) in the preceding 12 months referred to a weight management programme within 90 days of the BMI being recorded	9	7
Tests	31.	The patient has a cholesterol recorded in their medical record in the last 12 months	9	8
	32.	The patient has a heart/pulse recorded in their medical record in the last 12 months	9	8
	33.	The patient has a heart/pulse recorded in their medical record in the last 6 months	9	8
	34.	The patient has random blood glucose (≥11.1 mmol/L)/ fasting blood glucose (≥7.0 mmol/L) recorded in their medical record in the past 6 months for all adults patients who are 40+ years old and who are overweight (BMI > 25) or obese (BMI > 30)	9	8
	35.	The percentage of patients who were tested for the presence of protein in the urine by sending a urine sample for estimation of the albumin: creatinine ratio in the last 12 months	9	8
	36.	The percentage of patients with a new diagnosis of hypertension who have a record of a test for haematuria in the three months before or after the date of entry to the hypertension register	9	8
	37.	The percentage of patients with a new diagnosis of hypertension who have a record of urinary albumin: creatinine ratio test in the three months before or after the date of entry to the hypertension register	9	8
Patient intermediate outcomes	38.	% of patients with a BP of <140/90 mmHg with no adverse medicine reactions in patients who are in step 2 of the algorithm of hypertension management for patients already on medication every six months	9	8
	39.	% of patients with a BP of <140/90 mmHg with no adverse medicine reactions in patients who are in step 3 of the algorithm of hypertension management for patients already on medication	9	7
	40.	% of patients with a BP of <140/90 mmHg with no adverse medicine reactions in patients who are in step 4 of the algorithm of hypertension management for patients already on medication every six months	9	7
	41.	% of patients with a BP of <140/90 mmHg with no adverse medicine reactions in patients who are in step 5 of the algorithm of hypertension management for patients already on medication six monthly	9	7
	42.	% of patients with a BP of <140/90 mmHg with no adverse medicine reactions in patients who are in step 6 of the algorithm of hypertension management for patients already on medication six monthly	9	7
	43.	% of patients with a BP of <140/90 mmHg with no adverse medicine reactions in patients who are in step 7 of the algorithm of hypertension management for patients already on medication six monthly	9	7
Patient referrals	44.	Indicators on when and to whom referral might be made and why	9	9
	45.	% of pregnant patients who were referred to district hospital services because they had severe pre-eclampsia and imminent eclampsia	9	9
Practice/ facility indicators	46.	The percentage availability of core CVDs/ Hypertension drugs available	9	7

Key: *A* Appropriateness, *F* Feasibility

#### Patient blood pressure level

All four quality indicators which categorised blood pressure levels as normal, mild, moderate, and severe hypertension were rated appropriate; however, three were not seen as feasible and one as equivocal. An example of these indicators is ‘The % of patients in the practice/unit/facility who has blood pressure ≤140/90 mmHg’, which is seen as equivocal. The three indicator statements about the percentage of patients in the facility that have mild, moderate, and severe hypertension were though not rated as feasible.

#### Patient treatment

Most of the 17 indicator statements about the percentage of patients in different steps of the stepwise treatment without compelling indications, or with a specific condition such as stroke in the last 3 months, were rated appropriate, but in all the statements their feasibility was seen as equivocal. An example of these indicators is ‘The % of patients in the practice/unit/facility who are on the treatment of hypertension with angina in the last 3 months’.

## Discussion

The WHO, World Bank Group and Organisation for Economic Co-operation and Development have identified the five foundational elements critical to delivering quality health care services as being health care workers; health care facilities; medicines, devices, and other technologies; information systems; and financing [15]. It is imperative to develop and apply a tested hypertension management framework that is congruent to these foundational elements in South Africa as well as Africa as a whole, to enhance the potential utility of applying agreed indicators.

Substandard quality of care contributes to the global disease burden and unmet health needs in the population [1, 15]. The lack of data regarding the quality of care provided to patients with hypertension at the PHC level in South Africa is associated with the lack of processes and tools to measure the quality of care [30]. This is because sustained quality improvement within the current public health care system, and the provision of appropriate care, remains problematic in South Africa [13]. Consequently, it is important for the Ministry of Health in South Africa to strengthen existing quality improvement initiatives to reduce the rising burden of NCDs in the country.

Hypertension currently exerts a considerable health and economic burden on South Africa [13], with evidence that patients are currently not receiving the necessary evidence-based care in PHC facilities [5, 12]. The focus on monitoring patients with hypertension in South Africa is an imperative given the low levels of reported medication adherence [31], and the fact that one‑third of patients often do not receive all their antihypertensive medicines from PHC facilities due to supply chain issues and generally poor access to quality care [7, 12]. This is also important for achieving the aim of Universal Health Coverage and to reduce high morbidity and mortality due to hypertension in South Africa, which is a key target for the National Strategic Plan for NCDs [13].

We believe this is the first time quality indicators have been developed for use in primary care in South Africa, based on hypertension management guidelines. A multidisciplinary panel of experts, all involved in the day-to-day management of people with hypertension, and from different provinces of South Africa, were used in the development of possible indicators. The mixed sample of panellists resulted in a wide variation of relevant views to hypertension management, including authors' of the South African Hypertension Guidelines, to enhance the possible utility of considered indicators.

From 102 guideline-based quality indicators, 45.1% of the quality indicators were rated both appropriate and feasible with agreement to improve the management of patients with diagnosed hypertension at the PHC level in South Africa. The individual-level indicators are intended to build the data for the whole practice indicators. The number of patients in which the individual-level indicator was applied becomes the numerator for the practice indicators, whereas the total number of patients who were managed in the facility would become the denominator for the practice indicators.

Lifestyle and behavioural changes can lead to improved hypertension outcomes in mild hypertension without the need for antihypertensive treatment [32]. Even in resistant hypertension, it is important to maintain advice on a healthy lifestyle during drug therapy [32]. Lifestyle advice is also important as previous research reported that 16.6% of people with hypertension indicated that financial difficulties were the cause of challenges in accessing medicines [33]. This is particularly the case in low- and middle-income countries with high levels of patient co-payments where illness can have catastrophic consequences for families.

Monitoring intermediate outcomes in the management of hypertension is an important intervention in improving the quality of care and patient outcomes [11, 18, 19, 34]. They assist with increasing the number of patients who are at the treatment goal of a blood pressure <140/90 mmHg and with identifying those currently not yet at their treatment goal as well as informing future interventions [8].

Whilst the panel agreed that all the indicators under the dimension ‘blood pressure levels’ were appropriate, they also agreed that it was not feasible to determine the number or percentage of all patients with blood pressure above the target goal (≥140/90 mmHg) in the context of PHC in South Africa. Panellists were also equivocal on one of the indicators under this dimension, which was about determining the number or percentage of patients with controlled blood pressure (<140/90 mmHg). The decisions of the panellists on the indicators under the dimension of ‘blood pressure levels’ can be considered appropriate, considering that the panellists found all the indicators in the dimension of ‘intermediate outcome’ as appropriate and feasible.

All the indicators under the dimension ‘intermediate outcome’ concern the number or percentage of patients with a blood pressure of <140/90 mmHg with no adverse reactions in patients who are already on hypertension medication in different steps of the algorithm of hypertension management. The data on the indicators under the dimension ‘intermediate outcome’ can indirectly lead to the number of patients who are not at treatment target goals (blood pressure level). Consequently, the agreement of the panellists that the use of the indicators under the ‘blood pressure level’ is not feasible. Their decision may also have been based on the evidence that measuring quality requires measuring positive outcomes, count, or percentage of patients with a blood pressure of <140/90 mmHg in this case, and not vice versa [8].

The panel also found most of the indicators under the dimension ‘treatment’ and ‘blood pressure level’ appropriate but either with equivocal feasibility or not feasible. The main reason for the panellists not finding the indicators under the dimensions ‘treatment’ and ‘blood pressure level’ feasible was a concern about the data quality and precision needed in the recording of data, as data management is currently mostly manually and only partially electronic. The panellists also emphasised the need to use quality indicators as a means to assess the management of patients with hypertension, in the context of the RAND/UCLA Appropriateness Method focus on the ‘average’ patient, not those with specific details. Further work will be required to modify the indicators especially those encompassing adherence to the stepwise approach to the management of hypertension (indicators under ‘treatment’ dimension). Indicators rated appropriate and feasible under the dimension ‘referral’ may assist in identifying the data for which indicators under the dimensions ‘blood pressure levels’ and ‘treatment’ were intended for. Based on the assumption that guidelines in the management of hypertension at the PHC level are adhered to, the indicators under the dimension ‘referrals’ would provide the measure in which the indicators under ‘blood pressure level’ and ‘treatment’ dimensions were intended for.

Most of the indicators under the dimensions ‘monitoring’ and ‘review of patients’ were found appropriate and feasible. These indicators underpin the importance of recording activities and measurements that should be undertaken with the patient during the visit. Subsequently, provide the basis for quality of health care actions to be addressed in other dimensions. This is also important as it facilitates population level data collection with accurate data, which can then be used to inform future interventions needed to improve and sustain the quality of care at the PHC level in South Africa.

Most of the indicators under the dimension ‘tests’ were also found to be appropriate and feasible. This is imperative as these indicators are about recording the tests that are performed with patients.

### Conclusions and implications for patient care

Applying the care stated in these indicators would improve clinical outcomes and the quality of life for patients with a confirmed diagnosis of hypertension being treated at the PHC level in South Africa. As a result, help reduce avoidable harm, morbidity and mortality. In addition, contribute towards more effective and efficient use of scarce health care resources.

Consequently, our findings have implications not only for key stakeholder groups in South Africa to improve the care of patients with hypertension in the public system, but also across Africa.

Hypertension is the leading modifiable cardiovascular disease risk factor in South Africa. This study provides a possible solution to the current lack of quality measuring tools of health care provided to patients with hypertension at the PHC level through identifying 46 evidence-based quality indicators specifically tailor-made to suit South African public PHC level settings. The indicators and framework for hypertension management from this study can be replicated for the management of type 2 diabetes mellitus and other NCDs in South Africa, which is a priority of the National Department of Health [1].

Outcomes from consensus techniques have face or content validity but the next step of the ongoing indicator development and testing protocol will be to test the 46 developed hypertension quality indicators for their data feasibility, reliability, and validity. In addition, to determine what implementation strategies might be needed in terms of workforce, facilities and medicines supply to be able to apply the indicators at the PHC level in South Africa. Testing will also consider the clinimetric properties of the indicators to assess their value as precise measurement instruments and proxy indicators for quality assessment including the availability of routine data, variability, measurability, applicability and potential room for improvement [35] in addition to assessing the appropriateness of hypertension management in the context of routine PHC/ambulatory care practice in South Africa. The implementation strategies and the definition of these indicators would be clearly articulated based on the South African PHC settings in a follow-up publication after testing the indicators at the PHC level. Reliable information is the foundation of decision-making in health care and appropriate next clinical steps for people with confirmed NCDs [16].

## Summary

### What is known about quality of care for patients with hypertension

- Evidence-based guidelines for the management of hypertension are available [1, 4, 5, 23].
- No locally agreed indicators to measure the quality of care in the management of hypertension at the PHC level in South Africa [15].
- There are concerns with the quality of care and control of blood pressure amongst patients diagnosed with and managed for hypertension at the PHC level in South Africa [8, 13].

### What this study adds in the quality of care for patients with hypertension

- Provide appropriate and feasible quality indicators that can be tested for implementation to measure and track quality of care for hypertension at the PHC level.
- Lay foundation for future development of appropriate and feasible quality indicators for the management of other non-communicable diseases in Africa.
- Provide all stakeholders with a platform to agree on suitable measures of quality of care, making it easy for acceptability and implementation.

## Data Availability

Please note that additional data are available from the corresponding author on request.
